# Mood and Age Predict Cognitive Complaints in Memory Clinic Patients: A Machine‐Learning and Linear Modeling Approach

**DOI:** 10.1111/ene.70583

**Published:** 2026-04-22

**Authors:** Florian W. Sander, Marie Pittet, Valeria Manera, Christine Krebs, Esther Brill, Andrea Brioschi‐Guevara, Philippe Ryvlin, Joaquin A. Anguera, Adam Gazzaley, Philippe Robert, Stefan Klöppel, Jean‐François Démonet, Giulia Binarelli, Arseny A. Sokolov

**Affiliations:** ^1^ BrainCare@NeuroTech, Service Universitaire de Neuroréhabilitation (SUN), Département des Neurosciences Cliniques, Centre Hospitalier Universitaire Vaudois (CHUV) and Institution de Lavigny Lausanne Switzerland; ^2^ Laboratoire CoBTeK (Cognition Behaviour Technology) Université Côte d'Azur Nice France; ^3^ University Hospital of Old Age Psychiatry and Psychotherapy University of Bern Bern Switzerland; ^4^ Centre Leenaards de la Mémoire Centre Hospitalier Universitaire Vaudois Lausanne Switzerland; ^5^ NeuroDigital@NeuroTech, Service de Neurologie, Département des Neurosciences Cliniques Centre Hospitalier Universitaire Vaudois (CHUV) Lausanne Switzerland; ^6^ Neuroscape Center University of California San Francisco San Francisco California USA; ^7^ Department of Neurology University of California San Francisco San Francisco California USA; ^8^ Department of Psychiatry University of California San Francisco San Francisco California USA; ^9^ CreApolis JLNoisiez Fondation Biot ‐ Sophia Antipolis France

**Keywords:** Alzheimer's disease, cognitive complaints, machine learning, mood, serious videogames

## Abstract

**Introduction:**

Cognitive complaints are often considered early indicators of Alzheimer's disease (AD) and commonly lead to memory clinic consultations. Prior studies suggest stronger associations between cognitive complaints and mood than with objective cognition, but this interplay remains poorly understood. Using a machine learning–supported approach, we aimed to (1) identify key predictors of cognitive complaints, and (2) compare the value of gamified versus standard neuropsychological testing in detecting subtle deficits.

**Methods:**

In this international multi‐center study, 98 participants (57 females; mean age 71.9, range 55–86) from three memory clinics completed the Cognitive Failures Questionnaire (CFQ), mood and apathy questionnaires, the tablet‐based gamified Adaptive Cognitive Evaluation Explorer (ACE‐X), and standard neuropsychological tests. Predictors of CFQ scores were examined using elastic net regression and the Boruta algorithm, followed by linear mixed‐effects modeling.

**Results:**

Greater mood symptoms were associated with more cognitive complaints, whereas increasing age was linked to fewer complaints. Study center accounted for additional variance. The final model explained a substantial proportion of variance (conditional R^2^ = 0.48, marginal R^2^ = 0.33). Participants had lower z‐scores on ACE‐X compared to standard testing, but neither predicted the severity of cognitive complaints.

**Discussion:**

Mood and age were main predictors of cognitive complaints in memory clinic patients. Although ACE‐X yielded lower normative scores than standard tests, neither cognitive measure was linked to complaints. These findings highlight the importance of systematically assessing mood, adopting personalized approaches when evaluating subjective and objective cognition, and the potential value of gamified assessments for screening populations at risk of AD.

## Graphical abstract

Using a machine learning–supported approach across three international memory clinics (*n* = 98), mood and age emerged as the strongest predictors of self‐reported cognitive complaints. Objective cognitive performance, whether standard or gamified, showed no association with subjective complaints. These findings highlight the importance of assessing mood in memory clinic evaluations and suggest gamified assessments may offer enhanced sensitivity for detecting subtle cognitive deficits.

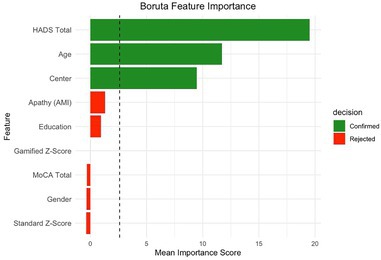

## 1 Introduction

Alzheimer's disease (AD) is a progressive neurodegenerative disease characterized by cognitive decline, behavioral changes, and impaired daily functioning. AD is the leading cause of major neurocognitive disorders (MND) [1], accounting for an estimated 60%–70% of cases worldwide according to the World Health Organization (*WHO Key Facts on Dementia*, published online on March 31 2025). Around the world, about 416 million people are estimated to fall within the AD continuum, representing up to 22% of people aged 50 and above [2].

AD pathology begins 10–20 years before the onset of clinical symptoms. When deficits are objectively detected, pathological changes such as amyloid‐beta plaque accumulation and tau tangles are usually already advanced [3, 4]. Therefore, current research efforts shift toward screening and preventive approaches, in order to enhance the effectiveness of interventions, including disease‐modifying treatments and neurorehabilitation, which could in turn positively impact prognosis and quality of life [5, 6].

Cognitive complaints are among the first clinical signs that appear and may represent a target for early screening and prevention of further decline [7]. The presence of cognitive complaints before the onset of formal objective cognitive deficits is defined as subjective cognitive decline (SCD) [8, 9]. SCD has shown moderate associations with AD biomarkers [7], and a meta‐analysis found that patients with SCD face a nearly two‐fold risk of developing MND compared to individuals without cognitive complaints [10]. Twenty percent of people with SCD convert to minor neurocognitive disorder, also labeled mild cognitive impairment (MCI) and 7% to MND [11]. Furthermore, patients with moderate to high levels of complaints are more likely to progress from SCD to MCI and MND [12].

The recently proposed SCD+ model identifies individuals with cognitive complaints and additional factors, such as subtle cognitive deficits or biomarker abnormalities as being at higher risk for developing AD [8]. Better differentiation and stratification of patients with SCD would allow for tailored management strategies, targeting those at a greater risk of progression. However, detecting cognitive decline in early screening of AD remains a significant challenge. Besides their unavailability to the general population, standard neuropsychological assessments are limited in capturing subtle decline in cognitive performance that may not result in formally deficient normative scores but represent a considerable loss of function for the affected individuals [10]. This is primarily due to the absence of baseline data as well as limited ecological validity [13]. Cognitive reserve may also account for the discrepancy between SCD and seemingly normal performance on standard cognitive assessments [14].

Digital assessments could offer a promising alternative. As opposed to neuropsychological testing, these tools are easily accessible (e.g., on a PC, phone, or tablet), do not require supervision and can easily be administered longitudinally [15, 16, 17]. Furthermore, gamified assessments provide an engaging and interactive environment and immediate feedback, which in turn potentially allows for a better mobilization of cognitive resources and more adequate representation of the multi‐modal cognitive challenges in everyday life [18, 19, 20, 21]. Yet, the application of gamified cognitive assessments for early AD screening remains understudied.

On the other hand, self‐reported mood symptoms appear to predict cognitive complaints more reliably than objective cognitive tests [22, 23]. A study in healthy individuals showed that Cognitive Failures Questionnaire (CFQ) scores exhibit moderate‐to‐strong positive associations with self‐reported depressive and anxious symptoms [24]. Depression, anxiety and apathy have also been identified as early features of AD pathology and are increasingly considered part of its initial clinical presentation [22, 23, 25, 26, 27]. Younger age and professional occupation have also been linked with higher cognitive complaints [28, 29]. However, the links between cognitive decline, cognitive complaints, demographic factors and mood in older people presenting for work‐up of neurocognitive disorders remain little understood.

In this international multi‐center observational study, we adopted a machine learning–supported framework to disentangle the contributions of mood, objective cognitive performance, and demographic factors to the severity of cognitive complaints as measured by the CFQ in older adults consulting a memory clinic. The principal aim of this study was to identify the most relevant predictors of cognitive complaints using a data‐driven feature selection framework.

As a secondary aim, we compared cognitive scores between the tablet‐based gamified Adaptive Cognitive Evaluation—Explorer (ACE‐X) and standard neuropsychological tests, and we evaluated whether gamified assessment may be a better predictor of the severity of cognitive complaints.

## 2 Methods

For the sake of conciseness, the full text and references for all assessments are available in the Supporting Informations.

### 2.1 Participants

This international multi‐centric study recruited patients presenting cognitive complaints from three memory centers: the Department for Geriatric Psychiatry and Psychotherapy in Bern (Switzerland), the Memory, Resources and Research Center in Nice (France), and the Leenaards Memory Center in Lausanne (Switzerland). Patients in all three centers were referred to memory clinics through routine clinical pathways (self‐referral, general practitioner, or specialist referral). All consecutive patients meeting the inclusion criteria during the study period were invited to participate. No additional selection beyond inclusion/exclusion criteria was applied.

Inclusion criteria were: (a) age > 55 years, (b) informed consent, (c) comprehension of instructions in French or German, (d) no major neurocognitive or psychiatric disorder according to the DSM V, (e) sensory‐motor ability to use a tablet, and (f) a Clinical Dementia Rating scale score < 1. Ethical approval was granted by the respective ethics committees for each center. All participants provided written informed consent.

### 2.2 Data Collection

Self‐reported cognitive complaints, mood, and apathy were assessed using three widely used patient reported outcome measures (PROMs), with higher scores indicating greater levels of complaints, and scores used as continuous variables in our analyses:

- The Cognitive Failures Questionnaire (CFQ) as a global measure of perceived cognitive difficulties.
- The Hospital Anxiety and Depression Scale (HADS) total score for “mood”, and the sub‐scores for anxiety and depression.
- The Apathy and Motivation Index (AMI) total score.

**FIGURE 1:**
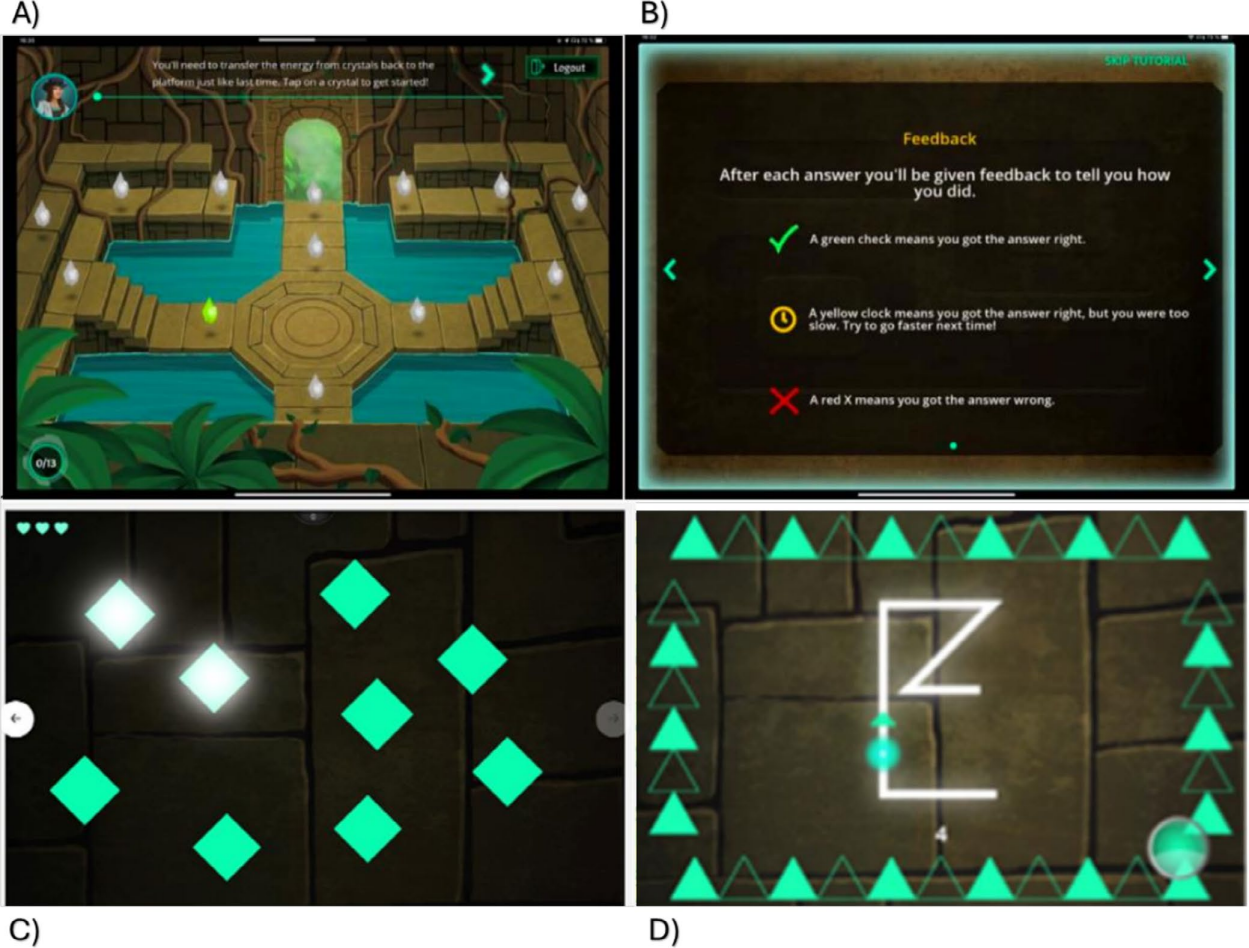
ACE‐X screenshots. (A) Home‐screen where each gem represents an assessment module. (B) Tutorial explaining the feedback indicators and their meanings. (C) In the Gem‐Chaser module, participants are required to memorize and recall the sequence of illuminated rhombi. (D) Dual task condition of the TNT module, requiring participants to simultaneously trace the shape on the center of the screen with the non‐dominant hand while selectively tapping on the circle at the bottom‐right of the screen when recognizing a green triangle pattern, and ignoring other geometric shapes appearing on the edges of the screen.

ACE‐X is a gamified, adaptive mobile cognitive test developed by the Neuroscape Center at the University of California San Francisco (UCSF) [16]. The administered ACE‐X modules included the Basic Reaction Time (BRT) task for response speed, Gem‐Chaser (forward and backward) for visuospatial short‐term and working memory, and the Tap'N'Trace (TNT) task for dual‐tasking performance. Figure 1 displays screenshots from the ACE‐X tests.

The standard neuropsychological battery was created to match the cognitive functions assessed by ACE‐X and included the Test of Attentional Performance (TAP) Alertness subtest for basic visual alertness, the WMS‐III Spatial Span (forward and backward) for visuospatial short‐term and working memory, and Baddeley's dual‐task assessment (digit recall and tracking tasks) to evaluate multitasking performance. We also included the Montreal Cognitive Assessment (MoCA) as a screening measure of global cognition.

All tests and PROMs were administered by trained clinicians on the same day. Socio‐demographic data (age, sex, education) were collected. Data were de‐identified and stored on secure online servers.

### 2.3 Statistical Analysis

All analyses were performed using R Software (v4.4.2; R Core Team 2024).

### 2.4 Data Pre‐Processing

The available predictors of CFQ scores were age, gender, education (ranked as per Swiss Federal Office of Statistics with 1 = compulsory education, 2 = upper secondary education: vocational education and training, 3 = upper secondary education: general education, 4 = tertiary education: professional education, and 5 = tertiary education: higher education), data acquisition center, mood (HADS total), apathy (AMI), and cognitive performance (mean composite Z‐scores for standard and gamified tests, and MoCA). Raw ACE‐X scores were converted to Z‐scores using UCSF norms (Supporting Information). French and German norms were used for scoring the performance on neuropsychological tests according to the linguistic zone of the respective centers. For ACE‐X and neuropsychological assessments, the Z‐scores for the specific tests were averaged across domains. Of note, TAP alertness data were only available for 17 patients (19%) in our final sample. The sample size for the primary analysis was not affected by missing TAP alertness values.

To assess the robustness of our approach, we re‐ran the entire analysis pipeline under several variations (e.g., entering HADS anxiety and depression subscales separately). These exploratory runs yielded comparable results, with no subscale consistently outperforming the total HADS score. For parsimony and interpretability, we therefore retained only the HADS total score in the final model. Participant characteristics were compared across centers with Kruskal‐Wallis (continuous) and χ^2^ tests (categorical). Normality was assessed via Q‐Q plots and boxplots; extreme values (> 3 SD) were removed.

### 2.5 Feature Selection

We included all available predictors of cognitive complaints within a parsimonious model. Feature selection was performed using machine learning. Elastic net regression was chosen for its ability to handle moderate correlations among predictors while performing variable selection through regularization [30]. A correlation matrix among PROMs and cognitive measures is included in the Supporting Information Table S2. This method combines Lasso (L1) and Ridge (L2) penalties, balancing model sparsity with predictive accuracy by shrinking weakly contributing coefficients toward zero. The model was trained on 70% of the data using 10‐fold cross‐validation to optimize λ (regularization strength) and α (mixing parameter). Performance was evaluated via RMSE and R^2^ on both training and test sets. We examined the stability of the regularized coefficients across cross‐validation folds to assess the robustness of each predictor. For descriptive purposes, we defined coefficients as “unstable” when their sign changed in more than 20% of folds or when amplitude varied markedly.

To validate and complement elastic net findings, the Boruta algorithm was applied [31]. Boruta is a wrapper method extending random forests that generates “shadow features” by permuting original predictors. Feature importance represented by Mean Decrease Accuracy (MDA) is compared to the highest‐scoring shadow. Significance is evaluated empirically through permutation‐based comparisons rather than traditional deterministic approaches. Predictors are classified as ‘confirmed’ if their importance is consistently higher than the maximum importance of shadow features across multiple permutations, ‘rejected’ if consistently lower, and ‘tentative’ if the difference is inconsistent. This empirical approach reduces false positives and ensures robust identification of variables most predictive of cognitive complaints, particularly in datasets with correlated predictors. The Boruta algorithm was recently used to obtain the most significant features for classification of AD [32]. Combining elastic net and Boruta strengthens the reliability and interpretability of our feature selection.

### 2.6 Final Model and Performance

Predictors selected by both elastic net and Boruta were included in a linear regression model for interpretability. Goodness‐of‐fit was evaluated using R^2^ (from the MuMIn package). Model performance was assessed by calculating within‐sample R^2^ using the entire dataset and a robust out‐of‐sample R^2^ using the separate test set.

### 2.7 Post Hoc Analyses

We conducted measures of uncertainty of our primary results by performing a nonparametric bootstrap with 1000 replicates on the final mixed‐effects model and derived percentile confidence intervals for each fixed effect. This approach provides a nonparametric estimate of the stability and precision of model coefficients, offering a more robust assessment of uncertainty given the modest sample size and potential heterogeneity across centers [33].

Given the multi‐center nature of our study and to assess generalizability of the outcomes, we conducted “Leave‐One‐Center‐Out” analyses by refitting the final mixed‐effects model three times, removing one center at each iteration. We examined whether the direction and magnitude of the associations for the selected variables and the CFQ scores remained consistent with the different iterations.

## 3 Results

### 3.1 Patients

A total of 98 patients were enrolled (Bern *N* = 55; Nice *N* = 26; Lausanne *N* = 17). Eight were excluded due to extreme predictor/outcome values (> 3 SD, *N* = 2) or incomplete data preventing Z‐score computation and elastic net model fitting (*N* = 6). Table 1 summarizes characteristics of the 90 retained patients by data acquisition center. Populations differed across centers in age (Lausanne younger), gender (more males in Lausanne), mood (lower depression and anxiety in Bern), apathy (lower apathy in Nice), and cognitive complaints (lower in Bern; all details including means and variances are provided in Table 1).

**TABLE 1:** Patients' characteristics across the three centers of data acquisition.

		Center				
		Lausanne	Bern	Nice	p‐value	Total
**Participants** ^†^	N	15	54	21		90
**Age** ^†^	Mean (SD)	**66.5 (5.6)**	72.9 (6.3)	73.4 (6.9)	0.002**	71.9 (6.7)
**Gender** ^‡^	Females (%)	**4 (26.7%)**	31 (57.4%)	16 (76.2%)	0.012*	51 (56.7%)
	Males (%)	**11 (73.3%)**	23 (42.6%)	5 (23.8%)		39 (43.3%)
**Education** ^‡^	lvl 2 (%)	2 (13.3%)	3 (5.5%)	0 (0.0%)	0.106	5 (5.6%)
	lvl 3 (%)	6 (40.0%)	27 (50.0%)	4 (19.0%)		37 (41.1%)
	lvl 4 (%)	2 (13.3%)	7 (13.0%)	4 (19.0%)		13 (14.4%)
	lvl 5 (%)	5 (33.3%)	17 (31.5%)	13 (61.9%)		35 (38.9%)
**Mean Z standard tests** ^†^	Mean (SD)	−0.7 (0.9)	−0.2 (0.7)	−0.3 (1.0)	0.146	−0.3 (0.8)
**Mean Z gamified tests** ^†^	Mean (SD)	−1.3 (1.5)	−0.9 (1.0)	−0.6 (1.2)	0.229	−0.96 (1.2)
**MoCA total**	Mean (SD)	26.5 (2.0)	25.7 (3.7)	25.0 (3.3)	0.429	25.7 (3.4)
**HADS total** ^†^	Mean (SD)	12.5 (7.6)	**7.9 (5.3)**	11.6 (5.0)	0.004**	9.5 (5.9)
**AMI total** ^†^	Mean (SD)	1.4 (0.3)	1.5 (0.5)	**1.1 (0.5)**	0.019*	1.4 (0.5)
**CFQ total** ^†^	Mean (SD)	40.9 (15.4)	**28.2 (10.2)**	43.1 (12.9)	< 0.001***	33.8 (13.6)

^†^ Kruskall‐Wallis test.

^‡^ Chi‐square test.

* p < 0.05.

** p < 0.01.

*** p < 0.001.

### 3.2 Elastic Net Regression Results

**FIGURE 2:**
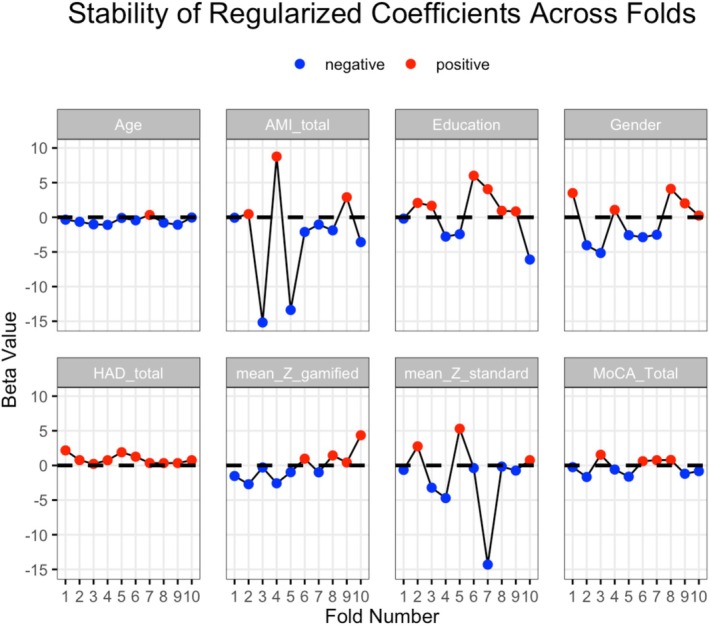
Stability of regularized regression coefficients across 10 cross‐validation folds for the available predictive parameters (center not included). Each subplot shows the beta values for a specific predictor (i.e., age) across the folds. Blue points represent negative coefficients, and red points represent positive coefficients. Greater observed variability (i.e., changes in sign in over 20% across folds or large amplitude differences) across folds indicates less stable contribution of the predictor to the model, while flatter lines indicate more stable coefficients.

The Elastic Net regression identified an optimal regularization strength (λ) of 3.16, and α = 0, i.e., the optimal model favored pure Ridge regression (Supporting Information Figure S1). All predictors were retained after regularization (Regularized coefficients for age = −0.54; AMI total = −1.93; center = 1.90; education = 1.34; gender = −2.28; HADS total = 1.01; mean Z gamified tests = −0.84; mean Z standard tests = 1.01; MoCA = −0.59). However, only HADS total scores and age showed stable coefficients (less than 20% of sign changes and no major variations in amplitude) throughout cross‐validation folds (Figure 2). Being multi‐categorical, the center variable could not be interpreted in the same way, as the sign of its coefficients (positive or negative) is not intuitive. Nevertheless, its high regularized coefficient suggests a stable association with CFQ scores. The elastic net model demonstrated moderate within‐sample performance on training data (*RMSE* = 10.03, *R* ^*2*^ = 0.47) and slightly reduced out‐of‐sample performance on test data (*RMSE* = 11.77, *R* ^*2*^ = 0.31), suggesting mild overfitting.

### 3.3 Boruta Results

**FIGURE 3:**
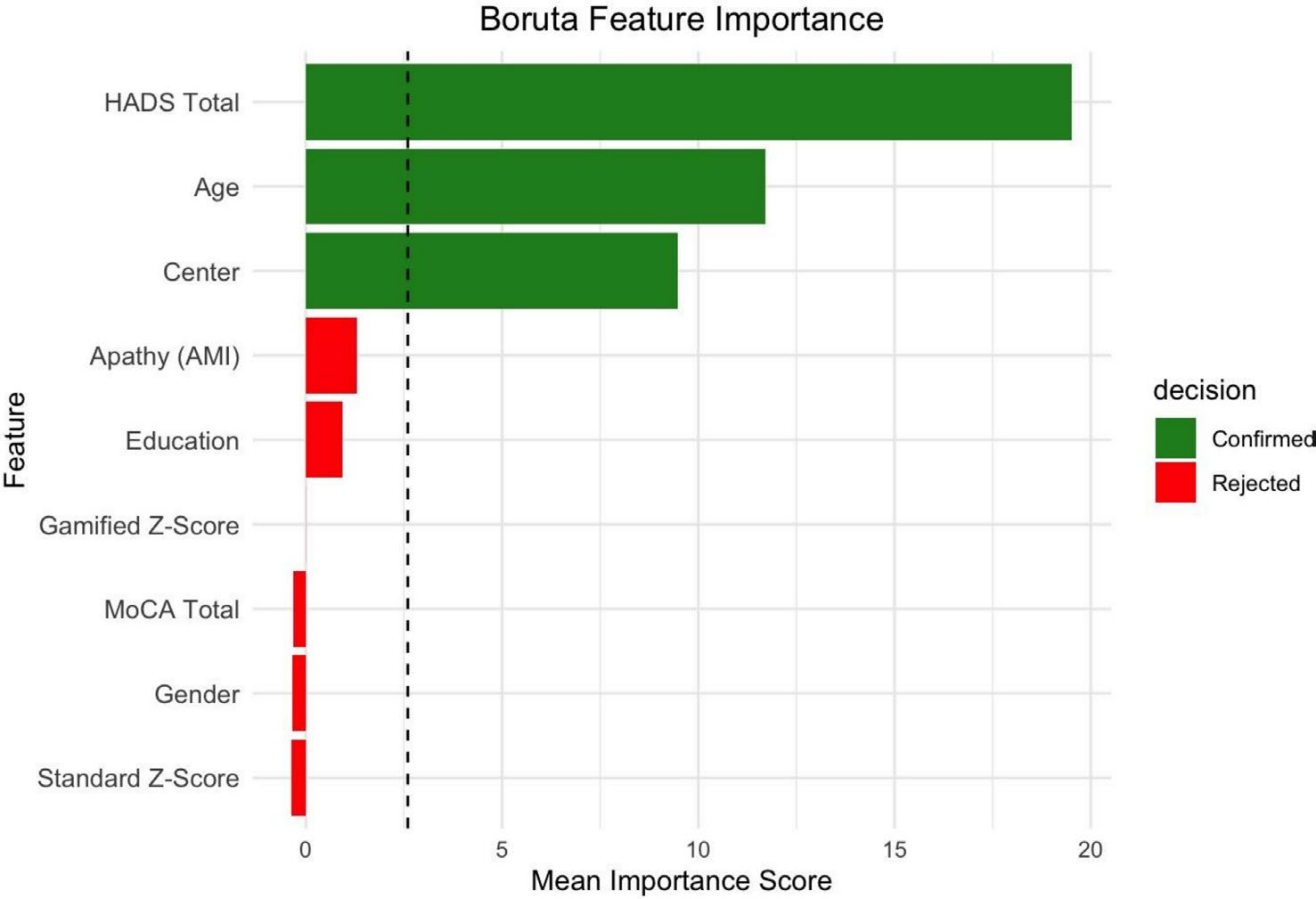
Feature importance ranking in predicting the CFQ score based on the Boruta algorithm. The mean importance scores for each predictor are shown. Features confirmed as important are displayed in green, while features rejected as unimportant are shown in red. The vertical dashed line indicates the maximum mean importance score among shadow (randomized) features.

The Boruta algorithm identified HADS total scores (*MDA* = 19.53), age (*MDA* = 11.72), and center (*MDA* = 9.47) as important predictors of cognitive complaints (Figure 3). Additionally, it confirmed the apparent unstable contribution of other factors according to the elastic net regression, by rejecting AMI total (*MDA* = 1.31), education (*MDA* = 0.93), mean gamified Z‐score (*MDA* = −0.004), MoCA total (*MDA* = −0.32), gender (*MDA* = −0.34), and mean standard neuropsychological testing Z‐score (*MDA* = −0.36). Of note, low positive MDA values indicate negligible contribution to explaining variance, whereas negative MDA values suggest that the variable may reduce model accuracy.

### 3.4 Final Linear Model

Given that the set of retained predictors included center—a variable suggesting a hierarchical data structure—we used a linear mixed model to account for varying intercepts across centers:

$$\mathrm{cfq}\_\text{total}\sim \text{HADS}\_\text{total}+\mathrm{age}+\left(1\;|\;\text{center}\right).$$

HADS scores were positively associated with CFQ scores, whereas age showed a negative association. Of note, there was no association between HADS and age (Supporting Information). Mean CFQ scores per center also significantly differed from each other (Table 1), justifying the inclusion of center as a random effect. This model explained a substantial proportion of within‐sample variance (conditional *R* ^*2*^ = 0.48, marginal *R* ^*2*^ = 0.33). Specifically, mood and age accounted for about a third of the variance (33%), while center contributed with an additional 15%. When applied to the test data, the model showed similar performance (conditional R^2^ = 0.43, marginal R^2^ = 0.34), thus demonstrating its robustness.

A leave‐one‐center‐out analysis showed that the association between mood (HADS) and cognitive complaints (CFQ) was preserved in all center combinations, indicating that the main findings were not driven by any single site. Age showed similar effects as in the main model, but slightly weaker and less consistent. Model explanatory power remained stable across iterations (marginal R^2^ = 0.21–0.45).

Bootstrap confidence intervals (1000 replicates) were computed to assess uncertainty around the fixed‐effect estimates. The bootstrap confirmed that mood was a robust positive predictor of cognitive complaints (95% CI: 0.84 to 1.72). In contrast, the confidence interval for age included zero (95% CI: −0.56 to 0.12), supporting its slightly weaker and more variable association. The intercept CI was wide, as expected in mixed‐effects models (95% CI: 13.30 to 64.43).

### 3.5 Comparison of Gamified With Standard Cognitive Assessment

The patients presented significantly lower normative performances on the ACE‐X as compared with the standard neuropsychological assessments (mean ACE‐X z‐score = −0.90; mean standard z‐score = −0.31; *p* < 0.001), suggesting that ACE‐X may have greater sensitivity when screening subtle cognitive deficits. However, performances on neither the gamified nor the standard cognitive assessments were retained as significant predictors of cognitive complaints in the analyses above, precluding further comparison. Sensitivity analyses varying the handling of the TAP alertness missing data (Supporting Information) yielded the same overall pattern of findings: mood and age consistently predicted cognitive complaints, while composite cognitive scores were not retained as relevant predictors.

## 4 Discussion

Taken together, we identify mood, age and center as significant predictors of self‐reported cognitive complaints in memory clinic patients, while objective cognitive performance on either gamified or standard neuropsychological assessments is not. We developed a holistic, data‐driven analytical framework to identify the strongest predictors of cognitive complaints. Unlike traditional approaches that rely on stepwise inclusion or arbitrary significance thresholds, our pipeline combines elastic net regularization and Boruta feature selection. Elastic net handles multicollinearity by shrinking correlated predictors and limiting overfitting, while Boruta compares each variable's importance against permuted “shadow” features to identify only those with stable, non‐random contributions. This dual approach therefore improves robustness, interpretability, and reduces the risk of false positives [30, 31].

To the best of our knowledge, only one multiple regression study using the Behavior Rating Inventory Executive Function—Adult version (BRIEF‐A) questionnaire in 1219 older people reported both a strong contribution of depression and anxiety as well as a moderate contribution of younger age to the severity of cognitive complaints (only on the problem‐solving index of the BRIEF‐A, for age) [29]. The present study is the first to formally quantify the relative predictive contributions of mood, age, and other clinical variables, including a gamified cognitive assessment, to the severity of cognitive complaints in memory clinic patients using a rigorous, data‐driven pipeline. This may be particularly relevant given the increasingly considered significance of SCD in the AD continuum [7, 8, 9, 10, 11].

Intriguingly, both in the community setting [24], and in the present international memory clinic cohort, older age appears to be associated with a lower magnitude of cognitive complaints. This may be due to various factors, such as greater cognitive challenges in the work environment, greater exposure and comparison to peers, professional and societal expectations, or higher exposure to daily stressors among younger individuals [34]. Daily life structure may also mask early deficits: older adults, particularly when retired, may face fewer demanding everyday tasks, making subtle changes harder to notice. In contrast, middle‐aged working individuals might detect changes earlier, even if their work demands do not solely explain the prevalence of complaints [28]. Furthermore, lower cognitive complaints in older adults may reflect age‐related shifts in expectations [35], normalization of decline, and reduced awareness of actual cognitive changes, potentially also due to greater social isolation [36] and thus a lack of external input. Finally, MND‐related stigma in older populations may lead to underreporting [37, 38]. This has two implications: first, structured assessments of insight and awareness should be included in clinical practice (perhaps even before addressing patients to memory clinics), especially for older individuals with minimal complaints. Discrepancy scores or performance prediction tasks can aid the detection of early anosognosia [39]. Second, the findings call for age‐sensitive screening strategies that consider how expectations, routine, mood, and stigma shape subjective appraisal of cognitive function. Clinicians should be vigilant for both excessive complaints in middle‐aged adults and a lack of complaints in older adults.

Our findings extend previous data on associations between mood and self‐reported cognitive deficits in community‐dwelling mid‐life and older individuals [22, 23, 25]. Consistent with these earlier reports, our models identify mood as a key statistical predictor of subjective complaints. Because both measures are subjective in nature, this relationship likely reflects shared perceptual and affective influences rather than a direct causal link. Systematic screening and comprehensive evaluation of mood disorders may therefore improve diagnostic accuracy and help contextualize subjective cognitive deficits. Individuals with both mood disorders and perceived cognitive deficits face a higher risk of progressing along the AD continuum [8, 22, 23, 25, 26, 40, 41]. Interestingly, amyloid pathology has been found to be associated with heightened awareness (hypernosognosia) and complaints of memory decline in individuals who do not present objective cognitive deficits [42]. While we cannot infer causality—whether mood alterations increase sensitivity to normal cognitive lapses or cognitive failures exacerbate mood symptoms, systematic screening and comprehensive evaluation of mood disorders in memory clinic consultations may help improve diagnostic accuracy and guide early interventions aimed at mitigating its impact.

Although mood disorders are common in SCD and early AD, the impact of treatment has rarely been assessed. Preliminary evidence is encouraging: the ASPIRE meta‐cognitive group intervention improved daily activity satisfaction and functioning in SCD and MCI patients [43], while a pilot combining mindfulness‐based stress reduction training with transcranial direct current stimulation yielded moderate effect sizes in reducing anxiety and depression, and a small effect size in improving objective cognition, though none of these differences reached statistical significance [44]. A recent review of SCD interventions also highlighted potential benefits of psychological and lifestyle interventions on well‐being, meta‐cognition, and objective cognition, though many results did not reach significance and overall evidence remains low [45]. A growing body of evidence suggests that multidomain lifestyle interventions—combining cognitive training, physical exercise, dietary changes, and psychological support—can enhance executive functions, psychological well‐being, and even brain connectivity in individuals with SCD [46, 47, 48]. Although most studies are short‐term and not powered to assess effects on progression to MND, improvements in cognition and mood are encouraging early indicators.

The significant influence of study center was expected, given the marked, statistically significant differences in patient characteristics between the centers. In particular, lower HADS and CFQ scores in Bern compared to Lausanne and Nice may reflect cultural or linguistic variations in symptom reporting, as well as methodological or situational differences between clinics. For instance, differing referral patterns may attract patients at distinct stages of cognitive decline or with varying cognitive profiles, leading to divergent baselines in mood and complaints. Cultural nuances could also shape how individuals from German‐ versus French‐speaking regions perceive and report symptoms. Recent guidelines for the translation and cross‐cultural adaptation of PROMs provide a framework to address these issues [49]. Overall, our findings underscore the need for context‐sensitive approaches to SCD screening and assessment, as a uniform framework may not adequately capture the lived experience of SCD across populations, cultures and stages of cognitive decline.

In contrast to previous studies, gender [50], education [51] and apathy [52, 53] were not associated with the severity of cognitive complaints in our sample. Our participants' generally high education levels and low apathy scores with restricted variance likely limited these variables' predictive power and generalizability. Furthermore, the apathy‐cognitive complaints relationship may be more prominent in populations at greater neurodegeneration risk [53], a subgroup not specifically represented in this study.

The significantly lower normative scores on the tablet‐based ACE‐X testing as compared to corresponding standard neuropsychological assessments may indicate greater sensitivity of ACE‐X to subtle deficits in alertness, visuo‐spatial short‐term and working memory, and multi‐tasking—underlining the potential value of ACE‐X as a screening instrument for AD‐related cognitive decline. While in agreement with concepts on the added value of adaptivity and gamification in cognitive screening and assessment [18, 19, 20, 21], additional research is needed to confirm this outcome, as the present study used US norms for ACE‐X and French and German norms for the neuropsychological assessments.

Furthermore, neither gamified nor standard cognitive assessment performances were significantly associated with the CFQ scores. Previous studies in memory clinic patients [54] and in multiple sclerosis [55] suggest that informant‐based reports on cognitive dysfunction align more closely with objective cognitive measures than self‐reported cognitive complaints. Optimized evaluation of cognitive complaints in older people may therefore rely on PROMs including self‐ and informant‐based reports, such as the Subjective Cognitive Decline Questionnaire (SCD‐Q) [56].

## 5 Limitations

Several limitations warrant consideration. First, more than 80% missing data for the TAP alertness task may have reduced the relevance of the standard neuropsychological composite z‐score. However, sensitivity analyses examining the impact of the TAP on the composite score did not change overall conclusions.

Moreover, the cognitive assessment was not exhaustive. Both the standard neuropsychological battery and the gamified ACE‐X battery primarily focused on cognitive control processes such as attention, working memory, and multitasking—rather than episodic memory that is most often associated with AD [57]. Nonetheless, cognitive control is critical for everyday functioning, with its deficiencies being linked to SCD and increased risk of later neurodegeneration [7]. Still, future studies should incorporate comprehensive episodic memory measures alongside executive and attentional tasks to better reflect the full spectrum of cognitive dysfunction in AD.

Furthermore, the modest sample size and considerable between‐center heterogeneity may limit generalizability. Differences in referral patterns and cultural or linguistic factors likely contributed to center effects, underscoring the need for replication in larger, more diverse cohorts. Our memory clinic sample also exhibited slightly higher levels of education than the regional average (Swiss Federal Office for Statistics).

Finally, while our study excluded patients with a CDR score ≥ 1 and those with a prior MND diagnosis, the data do not allow differentiating patients with SCD from those with MCI.

## 6 Future Perspectives

Future studies should clarify the interplay between cognition, mood, self‐awareness, and early neurodegeneration. Longitudinal and multimodal designs using robust analytical pipelines such as the one we present in this study are needed to distinguish factors underlying and resulting from cognitive complaints and highlight those predicting progression along the AD continuum. Although age and center were already significant predictors in our model, future studies with larger and more diverse samples are needed to more precisely characterize how age and cultural/linguistic context shape the reporting of cognitive complaints. Further work should refine the link between cognitive complaints and objective cognition, including more ecological and accessible tests for detecting subtle deficits. Additionally, rigorous trials are required to determine whether treating mood symptoms can both reduce cognitive complaints and slow disease progression in those at risk.

Finally, integrating novel biomarkers of AD and other neurodegenerative diseases with digital and gamified cognitive assessments may contribute to earlier and more accurate characterization of individuals at risk.

## Author Contributions

**Florian W. Sander:** conceptualization, writing – original draft, methodology, formal analysis, visualization, conceptualization, data curation. **Marie Pittet:** conceptualization, writing – original draft, methodology, visualization, formal analysis, software. **Valeria Manera:** conceptualization, investigation, writing – review and editing, data curation, project administration. **Christine Krebs:** project administration, investigation, data curation. **Esther Brill:** writing – review and editing, project administration, data curation, investigation. **Andrea Brioschi‐Guevara:** conceptualization, investigation, data curation, project administration. **Philippe Ryvlin:** writing – review and editing, supervision. **Joaquin A. Anguera:** software, writing – review and editing, data curation, resources. **Adam Gazzaley:** software, supervision, data curation, resources. **Philippe Robert:** conceptualization, investigation, writing – review and editing, project administration, data curation. **Stefan Klöppel:** conceptualization, investigation, writing – review and editing, project administration, data curation. **Jean‐François Démonet:** conceptualization, investigation, writing – review and editing, project administration, data curation. **Giulia Binarelli:** supervision, writing – review and editing, conceptualization, project administration. **Arseny A. Sokolov:** conceptualization, funding acquisition, writing – review and editing, validation, methodology, supervision, project administration.

## Funding

This work was supported by Stiftung Synapsis ‐ Alzheimer Forschung Schweiz AFS (Grant 2019‐CDA03).

## Conflicts of Interest

The authors declare no conflicts of interest.

## Supporting information


**Table S1.** Sensitivity analyses examining the impact of the TAP Alertness subtest on the standard composite score. Across all approaches (original composite with TAP, harmonized composite without TAP, complete‐case analysis, or adding a TAP‐inclusion indicator), the overall conclusions remained unchanged: mood (HADS) and age consistently predicted cognitive complaints, while cognitive composites were not retained as relevant predictors.
**Table S2.** Correlation Matrix of PROMs and domain‐level cognitive Z‐Scores. Stars: * *p* < 0.05, ** *p* < 0.01, *** *p* < 0.001.
Figure S1: Plot of the Elastic Net Performance. Our Elastic Net regression identified an optimal regularization strength (λ) of 3.16, and a mixing percentage α = 0, which means that the optimal model favored pure Ridge regression. RMSE: Root Mean Squared Error.

## Data Availability

The data that support the findings of this study are available on request from the corresponding author. The data are not publicly available due to privacy or ethical restrictions.

## References

[1] X. Li , X. Feng , X. Sun , N. Hou , F. Han , and Y. Liu , “Global, Regional, and National Burden of Alzheimer's Disease and Other Dementias, 1990–2019,” Frontiers in Aging Neuroscience 14 (2022): 937486, 10.3389/fnagi.2022.937486. 36299608 PMC9588915

[2] A. Gustavsson , N. Norton , T. Fast , et al., “Global Estimates on the Number of Persons Across the Alzheimer's Disease Continuum,” Alzheimer's & Dementia 19, no. 2 (2023): 658–670, 10.1002/alz.12694. 35652476

[3] V. L. Villemagne , S. Burnham , P. Bourgeat , et al., “Amyloid β Deposition, Neurodegeneration, and Cognitive Decline in Sporadic Alzheimer's Disease: A Prospective Cohort Study,” Lancet Neurology 12, no. 4 (2013): 357–367, 10.1016/S1474-4422(13)70044-9. 23477989

[4] H. Braak , D. R. Thal , E. Ghebremedhin , and K. Del Tredici , “Stages of the Pathologic Process in Alzheimer Disease: Age Categories From 1 to 100 Years,” Journal of Neuropathology and Experimental Neurology 70, no. 11 (2011): 960–969, 10.1097/NEN.0b013e318232a379. 22002422

[5] M. D. Mendonça , L. Alves , and P. Bugalho , “From Subjective Cognitive Complaints to Dementia: Who Is at Risk?: A Systematic Review,” Am J Alzheimers Dis Dementias 31, no. 2 (2016): 105–114, 10.1177/1533317515592331. PMC10852868 26142292

[6] C. H. van Dyck , C. J. Swanson , P. Aisen , et al., “Lecanemab in Early Alzheimer's Disease,” New England Journal of Medicine 388, no. 1 (2023): 9–21, 10.1056/NEJMoa2212948. 36449413

[7] S. Wolfsgruber , L. Kleineidam , J. Guski , et al., “Minor Neuropsychological Deficits in Patients With Subjective Cognitive Decline,” Neurology 95, no. 9 (2020): e1134–e1143, 10.1212/WNL.0000000000010142. 32636322

[8] F. Jessen , R. E. Amariglio , M. van Boxtel , et al., “A Conceptual Framework for Research on Subjective Cognitive Decline in Preclinical Alzheimer's Disease,” Alzheimer's & Dementia 10, no. 6 (2014): 844–852, 10.1016/j.jalz.2014.01.001. PMC4317324 24798886

[9] F. Jessen , R. E. Amariglio , R. F. Buckley , et al., “The Characterisation of Subjective Cognitive Decline,” Lancet Neurology 19, no. 3 (2020): 271–278, 10.1016/S1474-4422(19)30368-0. 31958406 PMC7062546

[10] K. E. Pike , M. G. Cavuoto , L. Li , B. J. Wright , and G. J. Kinsella , “Subjective Cognitive Decline: Level of Risk for Future Dementia and Mild Cognitive Impairment, a Meta‐Analysis of Longitudinal Studies,” Neuropsychology Review 32, no. 4 (2022): 703–735, 10.1007/s11065-021-09522-3. 34748154

[11] H. Li , C. C. Tan , L. Tan , and W. Xu , “Predictors of Cognitive Deterioration in Subjective Cognitive Decline: Evidence From Longitudinal Studies and Implications for SCD‐Plus Criteria,” Journal of Neurology, Neurosurgery, and Psychiatry 94, no. 10 (2023): 844–854, 10.1136/jnnp-2022-330246. 36868847

[12] A. X. Pereiro , S. Valladares‐Rodríguez , A. Felpete , et al., “Relevance of Complaint Severity in Predicting the Progression of Subjective Cognitive Decline and Mild Cognitive Impairment: A Machine Learning Approach,” Journal of Alzheimer's Disease 82, no. 3 (2021): 1229–1242, 10.3233/JAD-210334. 34151806

[13] N. Chaytor and M. Schmitter‐Edgecombe , “The Ecological Validity of Neuropsychological Tests: A Review of the Literature on Everyday Cognitive Skills,” Neuropsychology Review 13, no. 4 (2003): 181–197, 10.1023/B:NERV.0000009483.91468.fb. 15000225

[14] C. Pappalettera , C. Carrarini , F. Miraglia , F. Vecchio , and P. M. Rossini , “Cognitive Resilience/Reserve: Myth or Reality? A Review of Definitions and Measurement Methods,” Alzheimer's & Dementia 20, no. 5 (2024): 3567–3586, 10.1002/alz.13744. PMC11095447 38477378

[15] T. Parsons and T. Duffield , “Paradigm Shift Toward Digital Neuropsychology and High‐Dimensional Neuropsychological Assessments: Review,” Journal of Medical Internet Research 22, no. 12 (2020): e23777, 10.2196/23777. 33325829 PMC7773516

[16] K. D. O'Laughlin , B. H. Cheng , J. J. Volponi , et al., “Validation of an Adaptive Assessment of Executive Functions (Adaptive Cognitive Evaluation‐Explorer): Longitudinal and Cross‐Sectional Analyses of Cognitive Task Performance,” Journal of Medical Internet Research 27 (2025): e60041, 10.2196/60041. 40258271 PMC12053272

[17] K. L. Possin , T. Moskowitz , S. J. Erlhoff , et al., “The Brain Health Assessment for Detecting and Diagnosing Neurocognitive Disorders,” Journal of the American Geriatrics Society 66, no. 1 (2018): 150–156, 10.1111/jgs.15208. 29355911 PMC5889617

[18] E. Dulau , C. Botha‐Ravyse , M. Luimula , P. Markopoulos , and E. Markopoulos , “A Virtual Reality Game for Cognitive Impairment Screening in Elderly: A User Perspective” (2019), 10.1109/CogInfoCom47531.2019.9089973.

[19] W. Y. Hsu , W. Rowles , J. Anguera , et al., “Application of an Adaptive, Digital, Game‐Based Approach for Cognitive Assessment in Multiple Sclerosis: Observational Study,” Journal of Medical Internet Research 23, no. 1 (2021): e24356, 10.2196/24356. 33470940 PMC7840186

[20] A. A. Sokolov , A. Collignon , and M. Bieler‐Aeschlimann , “Serious Video Games and Virtual Reality for Prevention and Neurorehabilitation of Cognitive Decline Because of Aging and Neurodegeneration,” Current Opinion in Neurology 33, no. 2 (2020): 239–248, 10.1097/WCO.0000000000000791. 32073439

[21] T. Tong , M. Chignell , M. C. Tierney , and J. Lee , “A Serious Game for Clinical Assessment of Cognitive Status: Validation Study,” JMIR Serious Games 4, no. 1 (2016): e5006, 10.2196/games.5006. PMC4902858 27234145

[22] M. J. Slavin , H. Brodaty , N. A. Kochan , et al., “Prevalence and Predictors of “Subjective Cognitive Complaints” in the Sydney Memory and Ageing Study,” American Journal of Geriatric Psychiatry 18, no. 8 (2010): 701–710, 10.1097/JGP.0b013e3181df49fb. 21491631

[23] J. S. Siebert , T. Braun , and H. W. Wahl , “Change in Attitudes Toward Aging: Cognitive Complaints Matter More Than Objective Performance,” Psychology and Aging 35, no. 3 (2020): 357–368, 10.1037/pag0000451. 32134302

[24] Z. T. Goodman , K. R. Timpano , M. M. Llabre , and S. A. Bainter , “Revisiting the Factor Structure and Construct Validity of the Cognitive Failures Questionnaire,” Psychological Assessment 34, no. 7 (2022): 671–683, 10.1037/pas0001127. 35377689 PMC10044453

[25] R. Gallassi , A. Bisulli , F. Oppi , R. Poda , and C. Di Felice , “Subjective Cognitive Complaints, Neuropsychological Performance, Affective and Behavioural Symptoms in Non‐Demented Patients,” International Journal of Geriatric Psychiatry 23, no. 1 (2008): 95–101, 10.1002/gps.1901. 17879254

[26] G. M. MacQueen , S. Campbell , B. S. McEwen , et al., “Course of Illness, Hippocampal Function, and Hippocampal Volume in Major Depression,” Proceedings of the National Academy of Sciences 100, no. 3 (2003): 1387–1392, 10.1073/pnas.0337481100. PMC298782 12552118

[27] K. L. Lanctôt , Z. Ismail , K. K. Bawa , et al., “Distinguishing Apathy From Depression: A Review Differentiating the Behavioral, Neuroanatomic, and Treatment‐Related Aspects of Apathy From Depression in Neurocognitive Disorders,” International Journal of Geriatric Psychiatry 38, no. 2 (2023): e5882, 10.1002/gps.5882. 36739588 PMC10107127

[28] K. J. Rijs , T. N. V. den Kommer , H. C. Comijs , and D. J. H. Deeg , “Prevalence and Incidence of Memory Complaints in Employed Compared to Non‐Employed Aged 55–64 Years and the Role of Employment Characteristics,” PLoS One 10, no. 3 (2015): e0119192, 10.1371/journal.pone.0119192. 25742133 PMC4351065

[29] D. Smit , J. Koerts , D. Bangma , A. Fuermaier , L. Tucha , and O. Tucha , “Look Who Is Complaining: Psychological Factors Predicting Subjective Cognitive Complaints in a Large Community Sample of Older Adults,” Applied Neuropsychology. Adult 31, no. 3 (2024): 203–217, 10.1080/23279095.2021.2007387. 34882062

[30] H. Zou and T. Hastie , “Regularization and Variable Selection via the Elastic Net,” Journal of the Royal Statistical Society, Series B: Statistical Methodology 67, no. 2 (2005): 301–320, 10.1111/j.1467-9868.2005.00503.x.

[31] M. B. Kursa , A. Jankowski , and W. R. Rudnicki , “Boruta – A System for Feature Selection,” Fundamenta Informaticae 101, no. 4 (2010): 271–285, 10.3233/FI-2010-288.

[32] S. Ramu , N. Naik , and S. S. Bagalkot , “Boruta Feature Selection and Deep Learning for Alzheimer's Disease Classification,” International Journal of Statistics in Medical Research 14 (2025): 145–152, 10.6000/1929-6029.2025.14.15.

[33] N. Meinshausen and P. Bühlmann , “Stability Selection,” Journal of the Royal Statistical Society. Series B, Statistical Methodology 72, no. 4 (2010): 417–473, 10.1111/j.1467-9868.2010.00740.x.

[34] A. R. Stefaniak , J. M. Blaxton , and C. S. Bergeman , “Age Differences in Types and Perceptions of Daily Stress,” International Journal of Aging & Human Development 94, no. 2 (2022): 215–233, 10.1177/00914150211001588. 33739147

[35] N. Hill , S. Bhargava , J. Do , E. Bratlee‐Whitaker , and M. Brown , “Just as Expected? Older Adults' Aging Expectations Are Associated With Subjective Cognition,” Aging & Mental Health 29, no. 3 (2025): 444–451, 10.1080/13607863.2024.2399080. 39241125

[36] Z. Ran , J. Wei , G. Yang , and C. Yang , “Prevalence of Social Isolation in the Elderly: A Systematic Review and Meta‐Analysis,” Geriatric Nursing (London, England) 58 (2024): 87–97, 10.1016/j.gerinurse.2024.05.008. 38781629

[37] M. J. F. J. Vernooij‐Dassen , E. D. Moniz‐Cook , R. T. Woods , et al., “Factors Affecting Timely Recognition and Diagnosis of Dementia Across Europe: From Awareness to Stigma,” International Journal of Geriatric Psychiatry 20, no. 4 (2005): 377–386, 10.1002/gps.1302. 15799080

[38] L. Corner and J. Bond , “Being at Risk of Dementia: Fears and Anxieties of Older Adults,” Journal of Aging Studies 18, no. 2 (2004): 143–155, 10.1016/j.jaging.2004.01.007.

[39] S. F. Cappa , F. Ribaldi , C. Chicherio , and G. B. Frisoni , “Subjective Cognitive Decline: Memory Complaints, Cognitive Awareness, and Metacognition,” Alzheimer's & Dementia 20, no. 9 (2024): 6622–6631, 10.1002/alz.13905. PMC11497716 39051174

[40] S. A. Castellon , P. A. Ganz , J. E. Bower , L. Petersen , L. Abraham , and G. A. Greendale , “Neurocognitive Performance in Breast Cancer Survivors Exposed to Adjuvant Chemotherapy and Tamoxifen,” Journal of Clinical and Experimental Neuropsychology 26, no. 7 (2004): 955–969, 10.1080/13803390490510905. 15742545

[41] J. C. Scott , S. P. Woods , O. Vigil , et al., “A Neuropsychological Investigation of Multitasking in HIV Infection: Implications for Everyday Functioning,” Neuropsychology 25, no. 4 (2011): 511–519, 10.1037/a0022491. 21401259 PMC3125422

[42] P. Vannini , R. Amariglio , B. Hanseeuw , et al., “Memory Self‐Awareness in the Preclinical and Prodromal Stages of Alzheimer's Disease,” Neuropsychologia 99 (2017): 343–349, 10.1016/j.neuropsychologia.2017.04.002. 28385579 PMC5473166

[43] S. Rotenberg , N. D. Anderson , M. A. Binns , et al., “Effectiveness of a Meta‐Cognitive Group Intervention for Older Adults With Subjective Cognitive Decline or Mild Cognitive Impairment: The ASPIRE Randomized Controlled Trial,” Journal of Prevention of Alzheimer's Disease 11, no. 6 (2024): 1534–1548, 10.14283/jpad.2024.166. PMC11573849 39559867

[44] H. Brooks , H. A. Oughli , L. Kamel , et al., “Enhancing Cognition in Older Persons With Depression or Anxiety With a Combination of Mindfulness‐Based Stress Reduction (MBSR) and Transcranial Direct Current Stimulation (tDCS): Results of a Pilot Randomized Clinical Trial,” Mindfulness 12, no. 12 (2021): 3047–3059, 10.1007/s12671-021-01764-9. 34630733 PMC8491443

[45] R. Bhome , A. J. Berry , J. D. Huntley , and R. J. Howard , “Interventions for Subjective Cognitive Decline: Systematic Review and Meta‐Analysis,” BMJ Open 8, no. 7 (2018): e021610, 10.1136/bmjopen-2018-021610. PMC6059327 30030318

[46] C. Sheng , K. Yang , X. Wang , et al., “Advances in Non‐Pharmacological Interventions for Subjective Cognitive Decline: A Systematic Review and Meta‐Analysis,” Journal of Alzheimer's Disease 77, no. 2 (2020): 903–920, 10.3233/JAD-191295. 32741806

[47] E. Rolandi , A. Dodich , S. Mandelli , et al., “Targeting Brain Health in Subjective Cognitive Decline: Insights From a Multidomain Randomized Controlled Trial,” Aging Clinical and Experimental Research 37, no. 1 (2025): 151, 10.1007/s40520-025-03062-z. 40366507 PMC12078420

[48] A. Brioschi Guevara , M. Bieler , D. Altomare , et al., “Protocols for Cognitive Enhancement. A User Manual for Brain Health Services‐Part 5 of 6,” Alzheimer's Research & Therapy 13, no. 1 (2021): 172, 10.1186/s13195-021-00844-1. PMC8507160 34635149

[49] P. Cruchinho , M. D. López‐Franco , M. L. Capelas , et al., “Translation, Cross‐Cultural Adaptation, and Validation of Measurement Instruments: A Practical Guideline for Novice Researchers,” Journal of Multidisciplinary Healthcare 17 (2024): 2701–2728, 10.2147/JMDH.S419714. 38840704 PMC11151507

[50] M. D. Oliver , C. Morrison , F. Kamal , J. Graham , and M. Dadar , “Subjective Cognitive Decline Is a Better Marker for Future Cognitive Decline in Females Than in Males,” Alzheimer's Research & Therapy 14, no. 1 (2022): 197, 10.1186/s13195-022-01138-w. PMC9798694 36581949

[51] S. Arora , S. B. Patten , S. C. Mallo , et al., “The Influence of Education in Predicting Conversion From Subjective Cognitive Decline (SCD) to Objective Cognitive Impairment: A Systematic Review and Meta‐Analysis,” Ageing Research Reviews 101 (2024): 102487, 10.1016/j.arr.2024.102487. 39243892

[52] Q. Yang , Y. Wang , M. Yang , et al., “Apathy Co‐Occurs With Subjective Cognitive Decline Among Community‐Dwelling Older Adults,” Geriatric Nursing 48 (2022): 177–182, 10.1016/j.gerinurse.2022.09.018. 36257223

[53] A. Bogdan , R. Fabre , T. Desmidt , et al., “Different Trajectories of Apathy and Depression Among Subjective Cognitive Impairment Individuals With or Without Conversion to Dementia: Results From the Memento Cohort in France,” Journal of Alzheimer's Disease 95, no. 2 (2023): 415–426, 10.3233/JAD-230162. 37545236

[54] P. Regueira , I. Baldeiras , M. Lima , et al., “Disagreement Between Self‐ and Informant‐Reported Memory Complaints and Progression of Mild Cognitive Impairment to Dementia,” Aging & Mental Health 30, no. 1 106–115, 10.1080/13607863.2025.2552425. 40857141

[55] A. O'Brien , E. Gaudino‐Goering , M. Shawaryn , E. Komaroff , N. B. Moore , and J. DeLuca , “Relationship of the Multiple Sclerosis Neuropsychological Questionnaire (MSNQ) to Functional, Emotional, and Neuropsychological Outcomes,” Archives of Clinical Neuropsychology 22, no. 8 (2007): 933–948, 10.1016/j.acn.2007.07.002. 17851031

[56] L. Rami , M. A. Mollica , C. García‐Sanchez , et al., “The Subjective Cognitive Decline Questionnaire (SCD‐Q): A Validation Study,” Journal of Alzheimer's Disease 41, no. 2 (2014): 453–466, 10.3233/JAD-132027. 24625794

[57] D. Tromp , A. Dufour , S. Lithfous , T. Pebayle , and O. Després , “Episodic Memory in Normal Aging and Alzheimer Disease: Insights From Imaging and Behavioral Studies,” Ageing Research Reviews 24 (2015): 232–262, 10.1016/j.arr.2015.08.006. 26318058

