# Supplementary material for: Mood and Age Predict Cognitive Complaints in Memory Clinic Patients: A Machine‐Learning and Linear Modeling Approach
Source: Eur J Neurol. 2026 Apr 22;33(4):e70583. doi: 10.1111/ene.70583 (PMC13100495; doi:10.1111/ene.70583)
Supplement: Supplementary file 1 — Table S1. Sensitivity analyses examining the impact of the TAP Alertness subtest on the standard composite score. Across all approaches (original composite with TAP, harmonized composite without TAP, complete‐case analysis, or adding a TAP‐inclusion indicator), the overall conclusions remained unchanged: mood (HADS) and age consistently predicted cognitive complaints, while cognitive composites were not retained as relevant predictors. Table S2. Correlation Matrix of PROMs and domain‐level cognitive Z‐Scores. Stars: * p < 0.05, ** p < 0.01, *** p < 0.001. Figure S1: Plot of the Elastic Net Performance. Our Elastic Net regression identified an optimal regularization strength (λ) of 3.16, and a mixing percentage α = 0, which means that the optimal model favored pure Ridge regression. RMSE: Root Mean Squared Error. [file ENE-33-e70583-s001.docx]

Supplementary material

**Supplementary methods:**

**Standard neuropsychological test battery**

***General cognitive screening: the Montreal Cognitive Assessment, MoCA*** ^1^

The MoCA is a brief cognitive screening tool designed to detect mild cognitive impairment. It assesses multiple cognitive domains, including attention, executive function, memory, language, visuospatial abilities, and orientation. Scores range from 0 to 30 points, with a standard cutoff score < 26 suggesting cognitive impairment ^1^. A correction of +1 point is applied for individuals with ≤12 years of education. The MoCA is widely used in both clinical and research settings for early detection of cognitive deficits.

*Alertness: TAP alertness* ^2,3^

The Test of Attentional Performance (TAP) Alertness subtest is a computerized measure of basic alertness (reaction time). Participants are required to respond as quickly as possible to a visual stimulus. Reaction times and variability in responses are recorded as the outcome measures. The TAP alertness usually measures phasic alertness (triggered by a preceding auditory stimulus) as well, but we decided to focus on the visual aspect.

*Visuo-spatial memory: WMS-III spatial span, forward and backward modalities* ^4,5^

The Spatial Span subtest of the Wechsler Memory Scale—Third Edition (WMS-III) is a measure of visuospatial short-term and working memory. Participants are presented with a series of blocks on a board, which are designated by the evaluator in a specific sequence. In the forward condition, they reproduce the sequence in the same order, assessing short-term memory. In the backward condition, they recall the sequence in reverse order, evaluating working memory. The test begins with a sequence of two stimuli and progresses up to 9. Two main measures can be recorded for each condition: the total raw score (sum of correct answers) and the longest digit span. These measures are used to calculate a normative score for each condition (forward and backward span), which we used as our outcome measures.

*Multitasking: Baddeley / Brown-Peterson Dual task* ^6,7^

Participants completed a dual-task assessment combining digit recall and tracking tasks, performed both separately and simultaneously, based on Baddeley’s multitasking model and following Della Sala’s 2010 updated paper-and-pencil task ^6^.

Digit span was first determined by presenting verbal sequences of numbers (from 1 to 9), starting with two digits and increasing in length until participants could no longer correctly recall at least five out of six sequences. In the recall task, participants repeated sequences at their span length over 90 seconds, with accuracy measured by the proportion of correctly recalled digits.

The tracking task involved drawing a continuous line through 319 circles on an A3 sheet within 90 seconds, with performance measured by the number of circles crossed.

In the dual-task condition, participants performed both tasks simultaneously for 90 seconds. Performance was then assessed using proportional scores to evaluate dual-task interference (i.e. the ratio of circles crossed in the single vs dual task, and the ratio of the number of correct digit sequences in the single vs dual task).

**Gamified neuropsychological test battery**

***Adaptive Cognitive Evaluation Explorer, ACE-X*** ^8^

**ACE-X is an adaptive mobile cognitive test developed by the Neuroscape Center at the University of California San Francisco (UCSF). The tasks assess different aspects of cognitive functions (attention, working memory, and goal management), modified by incorporating adaptive algorithms, immersive graphics, video tutorials, motivating feedback, and a user-friendly interface. The adaptive algorithms allow each task to be completed in approximately 5 minutes, and ensure that comparisons between individuals of different ages, genders, races, or cultures reflect actual differences in their cognitive ability and not disparities in the testing parameters or ceiling/floor effects. ACE-X can also be used to track an individual’s changing cognitive control abilities over time.**

*Alertness: Basic Reaction Time (BRT)*

This task was designed to index the reaction time of participants on a simple task with minimal loading on executive function skills. Participants are instructed to detect the appearance of a symbol (target) by tapping a button each time the symbol appears. The target always appears in the center of the screen without distraction. Reaction times are measured for both index fingers. The outcome measure was the mean reaction time across both fingers between the appearance of the target on-screen and the correct button press.

*Visuo-spatial memory: Gem-Chaser forward and backward modalities*

The Forward Spatial Span task, based on the Corsi Block-Tapping Test ^5^, was designed to measure visuospatial short-term memory capacity. On each trial, participants view a test array of 9 green gems. In line with the typical administration of the original task, a sequence of gems is cued by consecutive changes in color of each gem involved in the sequence. Subsequently, the recall array is displayed, consisting of green gems arranged in the same way as the test array. Participants must recall the location of each cued gem in the order they were shown and indicate the location by tapping each cued location in the cued order. Participants begin the experimental task with a three-location sequence. Once the participant completes two consecutive trials of the previous level without error, they advance to the next level that includes an additional cued gem, thus increasing the difficulty. Participants complete as many levels as possible until two consecutive incorrect trials, at which point the task ends.

The Backward Spatial Span task was designed to measure visuospatial working memory. It follows the same design as the Forward Spatial Span task, but participants are instructed to revert the sequence in which the cued gems are presented in the test array.

The variable of interest for both modalities was the highest count of gems correctly recalled.

*Multitasking: Tap’N’Trace or Triangle Tracer*

The TNT task was designed to measure dual-tasking performance. Participants complete three conditions in sequential blocks. In the first block (‘tap only’ condition), the screen shows a frame of colored shapes (red, yellow, or green triangles, squares, or pentagons) and the participant is instructed to press a button with their dominant index finger only when the frame consists of green triangles (target) while ignoring all other colored shapes (distractors). In the second block, participants are instructed to trace a geometric shape in the center of the screen using their non-dominant hand (‘trace only’ condition). In the last condition (‘tap and trace’ condition), participants perform the first (tap) and second conditions (trace) simultaneously, tapping with their dominant finger and tracing with their non-dominant finger. The outcome of interest is the mean reaction time to correct trials in the ‘tap and trace’ condition.

***PROMs***

*The Cognitive Failures Questionnaire, CFQ* ^9^

The CFQ is a 25-item self-report questionnaire assessing the frequency of everyday cognitive lapses in attention, memory, and perception. Participants rate each item on a 5-point Likert scale (0 = never, 4 = very often), reflecting how often they experience specific cognitive failures (e.g., forgetting appointments, losing track of tasks). The total CFQ score ranges from 0 to 100, with higher scores indicating greater severity of cognitive complaints.

*The Apathy and Motivation Index, AMI* ^10^

The AMI is a 14-item self-report questionnaire measuring apathy and motivation across three domains: behavioral activation, emotional sensitivity, and social motivation. Participants rate each item on a 5-point Likert scale (0 = not at all, 4 = very much). Scores for each domain are calculated as the mean of the respective item ratings, and the total AMI score is the mean of all three domain scores. Lower scores indicate greater motivation, while higher scores reflect increased apathy (range from 0 – 4). A total score ≥ 1.91 indicates moderate apathy, and a score ≥ 2.38 severe apathy. We examined AMI scores as a continuous variable.

*The Hospital Anxiety and Depression Scale, HADS* ^11^

The HADS is a 14-item self-report questionnaire designed to assess symptoms of anxiety and depression in medical and general populations. It consists of two subscales: Anxiety (HADS-A) and Depression (HADS-D), each containing seven items rated on a 4-point Likert scale (0–3). Scores for each subscale range from 0 to 21, with higher scores indicating greater symptom severity. A score ≥11 in any of the two subscales indicates a clinical mood disorder. In this paper, we will refer to the total score of the HADS as “mood”. We examined total HADS score as a continuous variable.

**Statistical analysis**

***Data Pre-Processing***

All analyses were performed using the R Software (v4.4.2; R Core Team 2024).

The available variables for the prediction of cognitive complaints (CFQ scores) were the following: demographic variables (age, gender, education), the center of data acquisition (center with 3 levels: Lausanne, Bern, Nice), mood (HADS total scores), apathy levels (AMI scores), and cognitive performance (MoCA test scores, mean Z-score for gamified cognitive tests, mean Z-score for standard neuropsychological tests). Mean Z-scores for standard and gamified neuropsychological tests were obtained by converting raw scores to Z-scores using the available norms for each test. The norms for the ACE-X games were shared with us by the Neuroscape lab at UCSF ^8^. These Z-scores were then averaged across all standard cognitive tests, and across all gamified cognitive tests to obtain a composite measure of each. Of note, TAP alertness data were missing for 73/90 patients, thus the mean Z-scores of the standard assessments were less influenced by the construct of alertness less than the mean Z-scores of the gamified assessments.

Participant characteristics were compared across centers using Kruskal-Wallis tests for continuous variables and Χ² (chi-squared) tests for categorical variables. The normality of variable distributions was assessed through Quantile-Quantile (Q-Q) plots and boxplots. Extreme values (< 3 SD) were removed. To minimize the risk of overfitting, a train-test split was performed, where 70% of the data was randomly assigned for model training and the remaining 30% for model testing.

Feature Selection

Given the wide range of available variables, it was essential to identify and retain only the most relevant predictors through feature selection using machine-learning. We selected elastic net regression as our primary method, as it is well-suited for handling multicollinearity while performing variable selection through regularization^12^. However, in low-dimensional datasets with relatively few predictors and a moderate number of observations—as is the case here with 9 variables and 98 participants—elastic net may over-select features^13^. To validate and reinforce our findings, we therefore complemented this approach with the BORUTA algorithm, which applies a more conservative feature selection, although it is less optimal for datasets with correlated predictors^14,15^.

*Elastic net regression* ^12,13^

We designed an elastic net regression model predicting CFQ scores using all previously mentioned variables with the *caret* package. This type of regression with shrinkage methods is useful in the context of variable selection to optimize model efficiency by penalizing overcomplexity. The model combines standard Ordinary Least Square method with a mixture of Lasso (L1) and Ridge (L2) penalty. This combination balances model sparsity and predictive accuracy. Predictors weakly contributing to the prediction (coefficient shrunk to or near zero) are eliminated from the model.

Model fitting was done using a 10-fold cross-validation procedure on the training data. The optimized hyperparameters were $\lambda$, the strength of the regularization, and $\alpha$, the mixing parameter balancing L1 and L2 penalties. Model performance was assessed using Root Mean Square Error (RMSE) and the coefficient of determination (*R²*) on the training and test set (unseen data). To assess the relevance of all the predictors which survived elastic net shrinkage, we inspected the stability of their coefficients (i.e. direction and magnitude) through each fold of cross-validation.

*Confirmation with BORUTA algorithm* ^14,15^

We compared elastic net results with those of Boruta feature selection, a widely-used wrapper method extending random forests. The Boruta pipeline duplicates all predictor variables and randomly permutes them to create so-called “shadow features”, which have thus no association outcome. A random forest model is trained using both real predictors and shadow features. Each variable is permuted iteratively (including real predictors), and the model’s accuracy is recorded before and after each permutation. The Mean Decrease in Accuracy (MDA) is calculated as the difference in accuracy between the baseline and permuted data. The importance of each predictor is computed as the MDA between baseline and after permutation. Real predictors are then compared to the highest-scoring shadow feature: those with significantly higher importance are labeled as a “hit”. The number of hits is compared to the expected number of hits considering the number of iterations based on the binomial distribution. Only predictors with a number of hits significantly exceeding the expected value are retained in the final feature set.

*Final model and performance* ^16^

The predictors selected by elastic net shrinkage and Boruta feature selection were included in a linear regression model. A linear model was chosen for its interpretability, which is essential for clinical applications. The goodness-of-fit was evaluated using R², computed with the *MuMIn* package. The model’s performance was assessed in two ways: firstly, using the entire dataset to calculate within-sample R², and secondly, applying it to a separate test set (data not used in model fitting) to obtain a robust out-of-sample R² estimate.

**Post-hoc Analyses**

We conducted measures of uncertainty of our primary results by performing a nonparametric bootstrap with 1,000 replicates on the final mixed-effects model and derived percentile confidence intervals for each fixed effect. This approach provides a non-parametric estimate of the stability and precision of model coefficients, offering a more robust assessment of uncertainty given the modest sample size and potential heterogeneity across centers ^17^.

We also conducted “Leave-One-Center-Out” analyses by refitting the final mixed-effects model three times, removing one center at each iteration. We examined whether the direction and magnitude of the associations for the selected variables and the CFQ scores remained consistent with the different iterations.

**Additional results:**

We found no significant correlations between age and HADS Anxiety scores (ρ = - 0.170; p = 0.10), Depression scores (ρ = 0.013; p = 0.90) and total scores (ρ = - 0.083; p = 0.43).

| **Model** | **Predictors included** | **Key findings** | **R²m** | **R²c** | **Interpretation** |
| --- | --- | --- | --- | --- | --- |
| (A) Main model | Age, HADS, (1 \| Center) | Mood and age predicted CFQ | 0.33 | 0.48 | Core findings |
| (B) + Original standard composite (TAP heterogeneous) | + Standard composite | Composite not retained as relevant | 0.34 | 0.49 | No impact on conclusions |
| (C) + Harmonized composite (TAP excluded for all) | + Harmonized composite | Composite not retained as relevant | 0.34 | 0.49 | Robust to harmonization |
| (D) Complete-case (exclude 17 TAP-included cases) | + Harmonized composite | Composite not retained as relevant | 0.31 | 0.67 | Robust, lower N |
| (E) + TAP indicator | + TAP included (yes/no) | TAP flag not retained as relevant | 0.27 | 0.59 | No impact on conclusions |

*Supplementary table 1: Sensitivity analyses examining the impact of the TAP Alertness subtest on the standard composite score. Across all approaches (original composite with TAP, harmonized composite without TAP, complete-case analysis, or adding a TAP-inclusion indicator), the overall conclusions remained unchanged: mood (HADS) and age consistently predicted cognitive complaints, while cognitive composites were not retained as relevant predictors.*

**Supplementary Tables and Figures:**


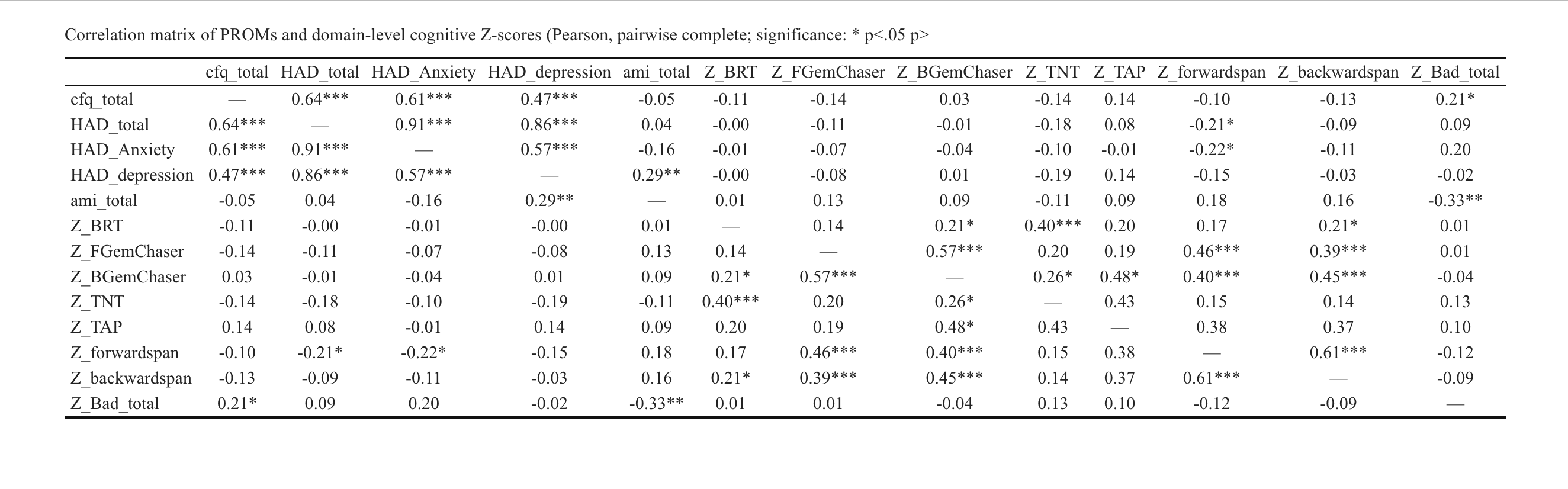


*Supplementary Table 2: Correlation Matrix of PROMs and domain-level cognitive Z-Scores. Stars: * p<.05, ** p<.01, *** p<.001.*


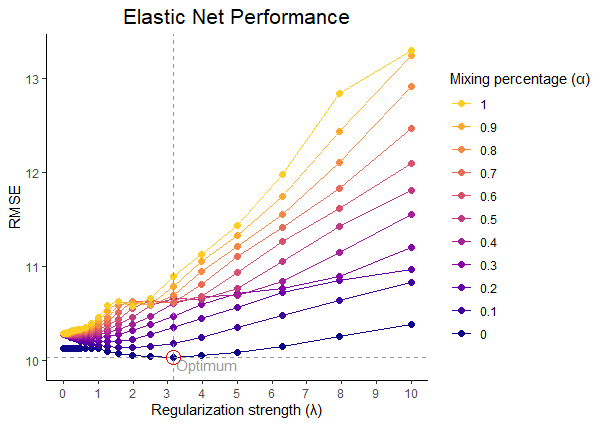


*Supplementary figure: Plot of the Elastic Net Performance. Our Elastic Net regression identified an optimal regularization strength (λ) of 3.16, and a mixing percentage* $\alpha$ *= 0, which means that the optimal model favored pure Ridge regression. RMSE: Root Mean Squared Error*

**Additional bibliography:**

1. Nasreddine ZS, Phillips NA, Bédirian V, et al. The Montreal Cognitive Assessment, MoCA: A Brief Screening Tool For Mild Cognitive Impairment. *J Am Geriatr Soc*. 2005;53(4):695-699. doi:10.1111/j.1532-5415.2005.53221.x

2. TAP - Test of Attentional Performance. Accessed March 11, 2025. https://www.psytest.net/en/test-batteries/tap/subtests

3. Becker M, Sturm W, Willmes K, Zimmermann P. Normierungsstudie zur Aufmerksamkeitstestbatterie (TAP) von Zimmermann, Fimm. *Z Für Neuropsychol ZNP*. Published online 1996.

4. Wiechmann A, Hall JR, O’Bryant SE. The Utility of the Spatial Span in a Clinical Geriatric Population. *Aging Neuropsychol Cogn*. 2010;18(1):56-63. doi:10.1080/13825585.2010.510556

5. Kessels RP, van Zandvoort MJ, Postma A, Kappelle LJ, de Haan EH. The Corsi Block-Tapping Task: standardization and normative data. *Appl Neuropsychol*. 2000;7(4):252-258. doi:10.1207/S15324826AN0704_8

6. Della Sala S, Foley JA, Beschin N, Allerhand M, Logie RH. Assessing Dual-Task Performance Using a Paper-and-Pencil Test: Normative Data. *Arch Clin Neuropsychol*. 2010;25(5):410-419. doi:10.1093/arclin/acq039

7. Eversheim U, Bock O. Evidence for processing stages in skill acquisition: A dual-task study. *Learn Mem*. 2001;8(4):183-189. doi:10.1101/lm.39301

8. O’Laughlin KD, Cheng BH, Volponi JJ, et al. Validation of an Adaptive Assessment of Executive Functions (Adaptive Cognitive Evaluation-Explorer): Longitudinal and Cross-Sectional Analyses of Cognitive Task Performance. *J Med Internet Res*. 2025;27:e60041. doi:10.2196/60041

9. Broadbent DE, Cooper PF, FitzGerald P, Parkes KR. The Cognitive Failures Questionnaire (CFQ) and its correlates. *Br J Clin Psychol*. 1982;21(1):1-16. doi:10.1111/j.2044-8260.1982.tb01421.x

10. Ang YS, Lockwood P, Apps MAJ, Muhammed K, Husain M. Distinct Subtypes of Apathy Revealed by the Apathy Motivation Index. *PLOS ONE*. 2017;12(1):e0169938. doi:10.1371/journal.pone.0169938

11. Zigmond AS, Snaith RP. The hospital anxiety and depression scale. *Acta Psychiatr Scand*. 1983;67(6):361-370. doi:10.1111/j.1600-0447.1983.tb09716.x

12. Zou H, Hastie T. Regularization and Variable Selection Via the Elastic Net. *J R Stat Soc Ser B Stat Methodol*. 2005;67(2):301-320. doi:10.1111/j.1467-9868.2005.00503.x

13. Liu L, Gao J, Beasley G, Jung SH. LASSO and Elastic Net Tend to Over-Select Features. *Mathematics*. 2023;11(17):3738. doi:10.3390/math11173738

14. Kursa MB, Rudnicki WR. Feature Selection with the Boruta Package. *J Stat Softw*. 2010;36:1-13. doi:10.18637/jss.v036.i11

15. Kursa MB, Jankowski A, Rudnicki WR. Boruta – A System for Feature Selection. *Fundam Informaticae*. 2010;101(4):271-285. doi:10.3233/FI-2010-288

16. Burnham KP, Anderson DR, eds. Statistical Theory and Numerical Results. In: *Model Selection and Multimodel Inference: A Practical Information-Theoretic Approach*. Springer; 2002:352-436. doi:10.1007/978-0-387-22456-5_7

17. Meinshausen N, Bühlmann P. Stability selection. *J R Stat Soc Ser B Stat Methodol*. 2010;72(4):417-473. doi:10.1111/j.1467-9868.2010.00740.x
